# Survivin but not Fms-like tyrosine kinase 3 ligand is up-regulated before the onset of rheumatoid arthritis: a pilot study

**DOI:** 10.1186/ar4474

**Published:** 2014-02-05

**Authors:** Maria Bokarewa, Mikael Brink, Malin Erlandsson, Solbritt Rantapää Dahlqvist

**Affiliations:** 1 Department of Rheumatology and Inflammation Research, Göregorg University, Box 480, SE-40530, Göteborg, Sweden; 2 Department of Public Health and Clinical Medicin/Rheumatology, Umeå University, SE-90185, Umeå, Sweden

## Abstract

**Introduction:**

Antibodies against citrullinated peptides (anti-CCP) and increased levels of cytokines precede the development of rheumatoid arthritis (RA) by several years. Recently, the proteins survivin and Fms-like tyrosine kinase 3 ligand (Flt3L) have been identified as biomarkers of RA associated with joint destruction. Our objective was to investigate the potential of survivin and Flt3L as predictors of RA in samples from patients prior to onset of symptoms.

**Methods:**

This study included 47 individuals sampled before onset of RA (median 2.5 years (IQR 4.5) and 155 matched controls, all were donors to the Medical Biobank of Northern Sweden, and 36 RA patients. Levels of anti-CCP, survivin and Flt3L were measured using ELISAs and 29 cytokines/chemokines by multiplex detection.

**Results:**

Levels of survivin were increased in pre-symptomatic individuals compared with controls (*P* = 0.003), whilst the levels of Flt3L were similar. The frequency of survivin positivity in the pre-symptomatic individuals was increased compared with the controls (36.2 vs.14.2%, *P* = 0.001) and predicted disease development (odds ratio (OR) =3.4 (95% confidence interval (CI) 1.6-7.2)). The frequency of survivin and Flt3L in RA patients was increased compared with the controls (both, *P* <0.0001, OR = 12.1 (95% CI, 5.3-27.6) and OR = 11.0 (95% CI, 3.9-30.9), respectively). Anti-CCP positive pre-symptomatic individuals and patients had significantly higher levels of survivin compared with anti-CCP2 negative individuals. In pre-symptomatic individuals, survivin correlated with IL-12, IL-1β and IL-9 whereas Flt3L correlated to a significantly broader spectrum of cytokines in RA patients.

**Conclusion:**

Proto-oncogene survivin was increased in individuals prior to onset of symptoms of RA and was correlated to cytokines suggesting its role at pre-clinical stages of the disease.

## Introduction

Rheumatoid arthritis (RA) is a chronic autoimmune disease characterized by inflammation of joint synovial tissue subsequently leading to the destruction of cartilage and bone. RA is considered a multifactorial disease with both genetic and environmental factors contributing to disease development [1-3]. However, the aetiopathogenic factors leading to disease development are not fully understood.

We, and others, have previously shown that anti-citrullinated protein antibodies of several fine specificities, as well as cytokines, can be detected several years before onset of disease, suggestive of an upregulation of immune system years before the onset of symptoms indicating a joint disease [4-8].

The ability to predict the development of RA in nonsymptomatic individuals is as yet limited, and therefore there is a need for additional biomarkers to be identified. Survivin is an intracellular protein with anti-apoptotic and cell-cycle regulatory functions, and Fms-like tyrosine kinase 3 ligand (Flt3L) is involved in the function of cells of the immune system [9,10]. High levels of survivin and Flt3L in blood and synovial fluid of patients with RA are implicated in the pathogenesis of joint inflammation [11-13]. Flt3L has recently been highlighted within a panel of preclinical biomarkers highly predictive for the development of RA [8].

In the present study, using blood samples from the Medical Biobank of Northern Sweden, we analyzed the levels of the two proteins, survivin and Flt3L, in presymptomatic individuals and matched controls together with samples taken at the time of diagnosis. The results were related to previously published concentrations of cytokines and chemokines [5].

## Methods

### Subjects

A case–control study was conducted within the purview of the Medical Biobank of Northern Sweden. The cohort is population based and all adult residents in the county of Västerbotten are continuously invited to participate. Collection of the blood samples and the storage conditions have previously been described in detail [6]. The register of patients fulfilling the 1987 American Rheumatism Association classification criteria for RA attending the Department of Rheumatology and with a known date for the onset of symptoms of joint disease was co-analyzed with the register of the Medical Biobank [14]. Forty-seven individuals were identified as being blood donors before the onset of symptoms (14 men and 33 females; prepatients), with a median predating (interquartile range) time of 2.5 (4.5) years before symptom onset. Control subjects were randomly selected from the same Medical Biobank cohorts as the predisease individuals, and were matched for sex and age at the time of blood sampling. A total of 155 control subjects (44 men and 111 women) were selected. Of the individuals identified as prepatients, 36 had also provided blood samples when attending the clinic at the time of diagnosis, with a median (interquartile range) time of 7.9 (5.2) months between onset of symptoms and the time of diagnosis (Table 1). The Regional Ethics Committee at the University Hospital, Umeå, Sweden approved this study, and all participants gave their written informed consent when donating samples.

**Table 1:** Demographic data of the presymptomatic individuals, rheumatoid arthritis patients and controls included in the study

	**Presymptomatic individuals (n = 47)**	**RA patients (n = 36)**	**Controls (n = 155)**
Females (%)	70.2	69.4	71.6
Median (interquartile range) age (years)	50.3 (18.7)	57.1 (7.6)	50.2 (17.1)
HLA-DR SE^a^, n (%)	28/47 (59.6)	24/34 (70.6)	43/105 (41.0)
Anti-CCP2 antibody-positive, n (%)	12/47 (25.5)	21/35 (60.0)	1/155 (0.6)

Anti-CCP, anti-cyclic citrullinated peptide antibodies; RA, rheumatoid arthritis. ^a^Shared epitope defined as human leukocyte antigen-DRB1 0101/0401/0404/0405/0408.

### Analysis of survivin and Flt3L concentrations

Survivin levels were measured in samples diluted 1:10 using a sandwich enzyme-linked immunoassay (ELISA) and a pair of matched antibodies (DY647; R&D Systems, Minneapolis, MN USA); the detection limit was set at 1 ng/ml. Flt3L levels were measured in undiluted samples with an ELISA using a pair of matched antibodies (DY308; R&D Systems), and a detection limit of 0.03 ng/ml. Cutoff levels for both survivin and Flt3L had previously been determined using samples from individuals with RA; samples containing >450 pg/ml survivin or >130 pg/ml Flt3L were considered positive [15].

### Analyses of anti-CCP antibodies and human leukocyte antigen shared epitope

Detection of anti-cyclic citrullinated peptide antibodies (anti-CCP) was performed using ELISAs according to the manufacturer’s instructions (Euro-Diagnostica AB, Malmö, Sweden). The cutoff value for positivity was set at 25 AU/ml according to the manufacturer.

Human leukocyte antigen-DRB1 genotyping for the 0101/0401/0404/0405/0408 alleles was performed as described previously [16].

### Analysis of cytokines, cytokine receptors and chemokines

The full methodology of cytokine analysis has been described previously [5]; briefly, 29 cytokines and chemokines were measured in plasma samples using multiplex detection kits from Bio-Rad (Hercules, CA, USA). The concentrations of interleukin (IL)-1β, IL-2, IL-4, IL-5, IL-6, IL-7, IL-8 (CXCL8), IL-9, IL-10, IL-12, IL-13, IL-15, IL-17, eotaxin (CCL11), IL-1 receptor antagonist, basic fibroblast growth factor, granulocyte colony-stimulating factor, granulocyte–macrophage colony-stimulating factor, interferon-gamma, interferon-inducible protein-10 (CXCL10), monocyte chemoattractant protein-1 (MCP-1/CCL2), macrophage inflammatory protein-1α (CCL3), macrophage inflammatory protein-1β (CCL4), platelet-derived growth factor BB, tumor necrosis factor alpha, vascular endothelial growth factor, monokine induced by interferon-gamma (CXCL9), macrophage migration inhibitory factor, and IL-2 receptor-alpha (CD25) in duplicate aliquots were analyzed with a Luminex 200 Labmap system (Luminex, Austin, TX, USA) as described previously [5]. Data analysis was performed using Bio-Plex Manager software version 4.1.1 (Bio-Rad). Standard curves were used to extrapolate the cytokine/chemokine concentrations.

### Statistical analysis

Continuous data were compared (prepatients vs. controls or vs. patients) by nonparametric analyses using the Kruskal–Wallis test for three groups and the Mann–Whitney test for two groups. The Spearman rank correlation test was used for correlation analyses. Logistic regression analyses were performed to identify associations between proteins and anti-CCP, respectively, for the development of RA and are presented as the odds ratio (OR) with the 95% confidence interval (CI). Relationships between categorical data (positive vs. negative) were compared using chi-square analyses. Considering the study to be explorative, *P* ≤0.05 was defined as being statistically significant. Statistical calculations were performed using the SPSS program for Windows, version 21 (IBM SPSS, Chicago, IL, USA).

## Results

### Levels of survivin and Flt3L in pre-symptomatic individuals, RA patients and controls

**Figure 1:**
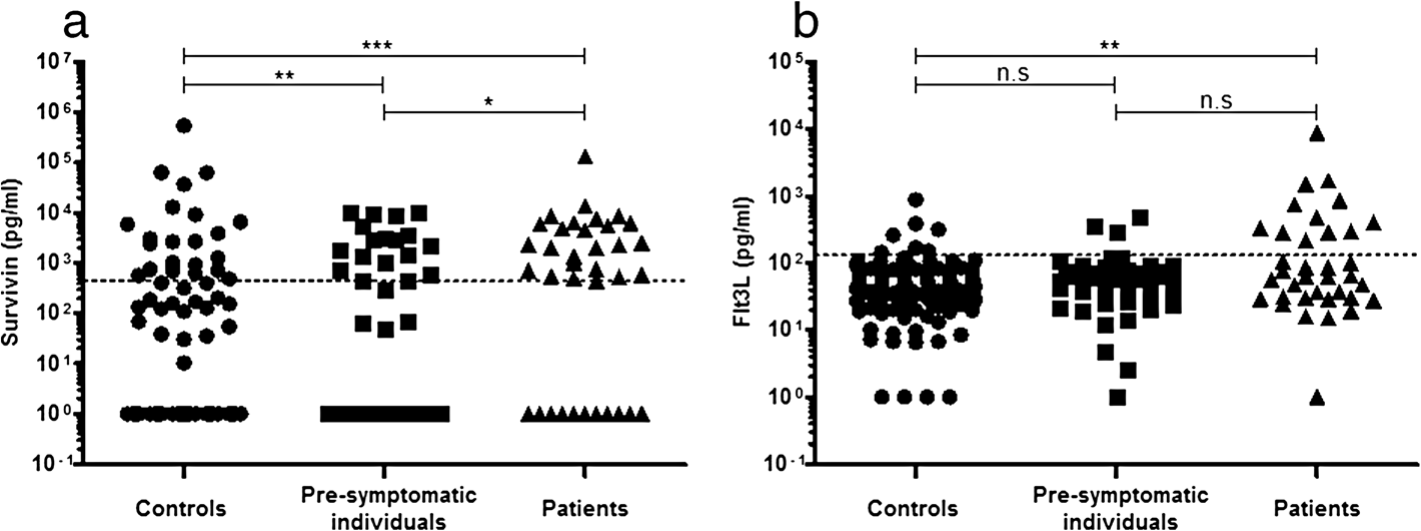
**Concentrations of survivin and Fms-like tyrosine kinase 3 ligand measured by enzyme-linked immunosorbent assay. (a)** Survivin concentration in controls (*n* = 155), presymptomatic individuals (*n* = 47) and patients with rheumatoid arthritis (RA; *n* = 36). A significant Kruskal–Wallis test was found between the three groups (*P* < 0.001). The Mann–Whitney U test was used for comparisons between two groups. ***P < 0.001, **P < 0.01 and *P < 0.05. **(b)** Fms-like tyrosine kinase 3 ligand (Flt3L) concentration in controls (*n* = 155), presymptomatic individuals (*n* = 47) and patients with RA (*n* = 36). A significant Kruskal–Wallis test was found between the three groups (*P* < 0.05). The Mann–Whitney U test was used for comparisons between two groups, **P < 0.01. n.s, not significant.

**Figure 2:**
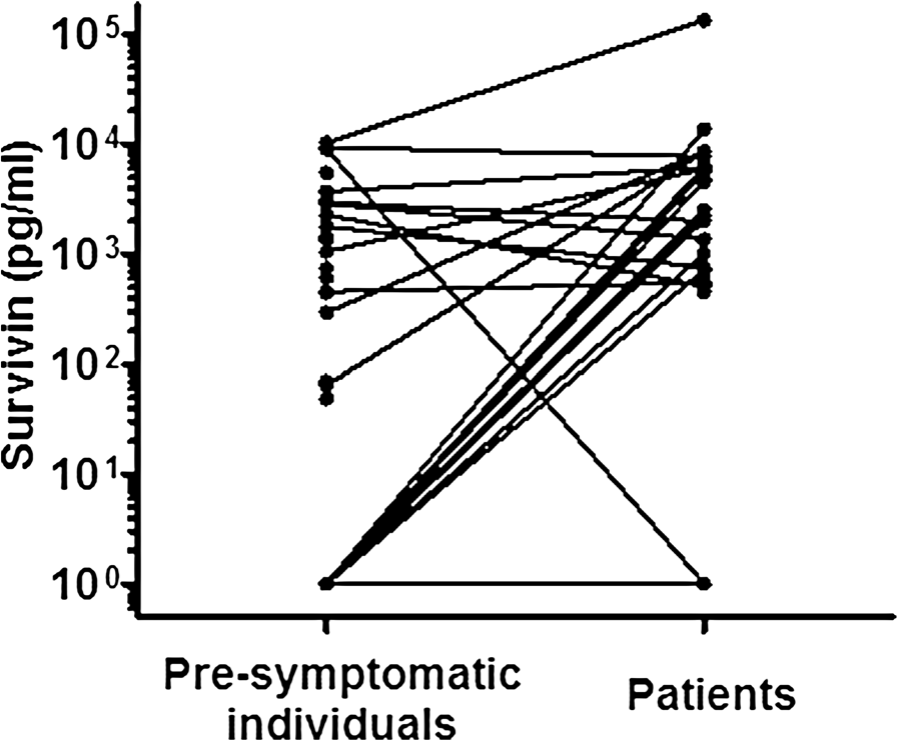
**Concentrations of survivin in presymptomatic individuals and rheumatoid arthritis patients.** A line indicates the same individual.

Analyzing the levels of survivin and Flt3L in plasma samples using a sandwich ELISA method showed that the concentrations of both survivin and Flt3L differed significantly between the three groups (*P* < 0.001 and *P* < 0.05, respectively). Survivin was significantly increased in the presymptomatic individuals compared with the controls (*P* = 0.003) whilst the levels of Flt3L (*P* = 0.21) were similar in presymptomatic individuals and controls (Figure 1a,b). The concentration of survivin increased significantly in RA patients compared with the presymptomatic individuals (Figures 1a and 2). There was no direct relationship between the levels of survivin and Flt3L and the time interval before the onset of the disease. However, the time interval for the presymptomatic individuals positive for survivin was closer to symptom onset compared with survivin-negative individuals (median (interquartile range), 1.36 (2.0) years and 4.10 (5.86) years, respectively, *P* = 0.012). The levels of survivin and Flt3L were significantly increased in RA patients compared with controls (*P* < 0.0001 and *P* = 0.005, respectively) (Figure 1a,b).

### Frequencies of survivin and Flt3L in presymptomatic individuals, RA patients and controls

The frequencies of survivin and Flt3L above respective cutoff values in controls, presymptomatic individuals and RA patients are presented in Table 2. The frequency of survivin in the presymptomatic individuals was significantly higher compared with controls (36.2% vs. 14.2%, *P* = 0.001). The OR for predicting disease in the presymptomatic individuals compared with controls was 3.4 (95% CI = 1.6 to 7.2) for survivin. The difference in the frequencies for RA patients compared with controls was found to be significant for both survivin and Flt3L (*P* < 0.0001, Pearson chi-square) with an OR of 12.1 (95% CI = 5.3 to 27.6) and 11.0 (95% CI = 3.9 to 30.9), respectively, for predicting disease.

**Table 2:** Sensitivity (%) and specificity (%) for presymptomatic individuals and rheumatoid arthritis patients. Odds ratio with 95% confidence interval, PPV and NPV calculated for presymptomatic individuals

	**RA patients (%)**	**Pre RA (%)**	**Specificity (%)**	**OR**^**a**^	**95% CI**^**a**^	***P *** **value**^**a**^	**PPV (%)**	**NPV (%)**
Survivin	63.9	36.2	88.3	3.43	1.62-7.23	0.0008	43.6	81.6
Flt3L	33.3	6.4	95.5	1.43	0.36-5.77	0.61374	30.0	77.1

CI, confidence interval; Flt3L, Fms-like tyrosine kinase 3 ligand; NPV, negative predictive value calculated for the presymptomatic individuals; OR, odds ratio; PPV, positive predictive value calculated for the presymptomatic individuals; RA, rheumatoid arthritis. ^a^Calculated for presymptomatic individuals compared with controls.

### Levels and frequency of survivin and Flt3L in relation to anti-CCP antibodies

Both presymptomatic individuals and RA patients who were positive for anti-CCP antibodies had higher levels of survivin (respectively *P* = 0.023 and *P* = 0.021, Mann–Whitney U test) when compared with anti-CCP negative individuals.

Among presymptomatic individuals the frequency of anti-CCP was 25.5%. There was a significant relationship between the level of survivin above the cutoff value (that is, being positive) and the presence of anti-CCP antibodies, 66.7% versus 33.3% below the cutoff value for survivin (χ^2^ = 6.49, *P* = 0.016). In multiple regression analysis including anti-CCP antibodies and survivin, the positivity for survivin remained a borderline significant predictor for disease development (OR = 2.26, 95% CI = 0.96 to 5.32, *P* = 0.061). In RA patients expressing anti-CCP antibodies, 85.0% had a level of survivin above the cutoff value compared with 15.0% being survivin-negative (χ^2^ = 5.62, *P* = 0.026).

There were no significant relationships between Flt3L concentration or positivity and presence of anti-CCP antibodies.

### Levels of survivin and Flt3L in relation to levels of cytokines/cytokine receptors and chemokines

The levels of survivin and Flt3L displayed significant correlation with a number of cytokines and/or chemokines analyzed in the samples from presymptomatic individuals and patients with RA. After correction for the number of comparisons, the levels of both survivin and Flt3L in the presymptomatic individuals correlated with the levels of IL-2, IL-9 and IL-12 (Table 3). For survivin, the pattern of correlations differed between the presymptomatic individuals and the RA patients. In the presymptomatic individuals, survivin correlated with more cytokines (IL-9, IL-12, IL-1β, IL-2, granulocyte–macrophage colony-stimulating factor) and these correlations were at a higher significance level. In the presymptomatic individuals, survivin was associated with the cytokines regulating formation of effector T cells (IL-2) with the major difference occurring in IL-9 and IL-12; that is, the cytokines predisposing and assuring formation of T-helper type (Th) 1 and Th17 proinflammatory T-cell subsets. After correcting for the number of comparisons, the positivity for survivin was only related to increased concentration of IL-9 (data not shown, *P* < 0.05). In RA patients, the correlations with survivin were changed from the regulatory cytokines to the proinflammatory cytokines produced by established Th subsets and to angiogenic factors; that is, interferon-gamma, tumor necrosis factor alpha, vascular endothelial growth factor and platelet-derived growth factor BB.

**Table 3:** Correlations between the concentrations of Survivin and a cytokine/chemokine

**Cytokines/chemokines**	**Presymptomatic individual**	**Patient**
Interleukin-12	0.592***	0.378
Interleukin-1β	0.559**	0.406
Interleukin-9	0.547**	0.573**
GM-CSF	0.535**	0.441
Interleukin-2	0.491*	0.420
Eotaxin	0.392	0.525*
Interleukin-4	0.369	0.544*
MCP-1	0.026	0.546*

Presented in descending order after Spearman correlation in presymptomatic individuals. Only cytokines that remained significant after multiple testing are presented. GM-CSF, granulocyte–macrophage colony-stimulating factor; MCP-1, monocyte chemoattractant protein-1. *P < 0.05, **P < 0.01 and ***P < 0.001 after adjustments for multiple comparisons.

The association between the cytokine/chemokine panel and Flt3L was different from that of survivin. Flt3L in presymptomatic individuals was associated with a broader cytokine pattern (Table 4) that became even broader after the onset of disease. The statistical associations found between cytokines and Flt3L in the presymptomatic individuals become stronger in the RA patients and remained significant after correction for multiple comparisons (Table 4). In RA patients, the positivity for Flt3L was associated with IL-13, IL-1β, granulocyte–macrophage colony-stimulating factor, IL-1 receptor agonist, IL-6, and IL-9 (*P* < 0.01 for all) and with IL-2 and IL-10 (*P* < 0.05 for both) after corrections for multiple comparisons. No association of positivity for Flt3L and cytokines/chemokines remained significant in the presymptomatic individuals (data not shown).

**Table 4:** Correlations between the concentrations of Flt3L and a cytokine/chemokine

**Cytokine/chemokine**	**Presymptomatic individual**	**Patient**
Interleukin-12	0.585***	0.515
Interleukin-2	0.507**	0.587**
TNFα	0.503**	0.527*
Interleukin-9	0.498*	0.72***
Eotaxin	0.497*	0.523*
Interleukin-10	0.449*	0.557*
IL-1Ra	0.445*	0.605**
Interleukin-1β	0.391	0.695***
GM-CSF	0.380	0.582**
Interleukin-13	0.301	0.595**
Interleukin-6	0.248	0.671***
MCP-1	0.131	0.625**
MIG	0.104	0.566*

Presented in descending order after Spearman Correlation in presymptomatic individuals. Only cytokines that remained significant after multiple testing are presented. IL-1Ra, interleukin-1 receptor antagonist; GM-CSF, granulocyte–macrophage colony-stimulating factor; MCP-1, monocyte chemoattractant protein-1; MIG, monokine induced by interferon-gamma; TNFα, tumor necrosis factor alpha. *P < 0.05, **P < 0.01 and ***P < 0.001 after adjustments for multiple comparisons.

## Discussion

In this pilot study we investigated the presence of survivin and Flt3L in plasma samples from presymptomatic individuals, population controls and RA patients collected at the time of diagnosis. Survivin and Flt3L have recently emerged as biomarkers of joint damage and poor response to anti-rheumatic treatment [17,18]. The present study addressed the question of whether the levels of these proteins were altered before the onset of RA symptoms, and whether they were associated with other inflammatory markers; for example, cytokines, chemokines and anti-CCP2 antibodies analyzed at the same time points. The levels of survivin in the samples collected years before onset of RA symptoms were significantly increased compared with matched controls. The levels of survivin were prominently high in the anti-CCP-positive presymptomatic individuals. In the these individuals, survivin was most strongly associated with IL-9 and IL-12 (that is, the cytokines predisposing and assuring formation of Th1 and Th17 proinflammatory T-cell subsets) and also IL-2, the cytokine regulating the formation of effector T cells. The concentration of Flt3L was most evidently increased in RA patients and to a lower extent in the presymptomatic individuals. In RA patients, the levels of Flt3L correlated to the cytokines of the Th1, Th2 and regulatory T-cell lineages and monocyte chemoattractant protein-1, known among the major chemoattractants produced by the inflamed synovia.

Survivin has been shown to be crucial at the early stages of T-cell development, where it is required for the formation of a functional T-cell receptor [19,20]. Survivin is also suggested important for intracellular transfer of signals from the co-stimulatory molecules during T-cell activation [21], and for the formation of memory immune responses [22]. In the context of arthritis, extracellular survivin has been found in the blood and synovial fluid of RA patients [11,23]. The proportion of survivin-positive patients varied between 60% in patients with early RA and 28% in a cohort of established and treated RA patients [11,13]. A prospective study of the Swedish patient cohort with early RA showed that high levels of survivin measured at the first visit to a rheumatologist were predictive for a severe cause of the disease [13]. Indeed, extracellular survivin was consistently associated with the development and progression of joint damage in RA and also distinguished patients with a therapy-resistant disease, nonresponders to biological treatment and those with a low rate of disease remission [11,13,18,23]. High levels of survivin were also detected in the rheumatoid synovia where the expression of survivin correlated with the synovial infiltration with macrophages and memory T cells and with the low rate of apoptosis. These findings suggested a key function for survivin in the regulation of invasive properties of fibroblasts in the inflamed rheumatic joint [24-26]. The result of our study is not consistent with these findings, since the concentration of survivin was increased before the patients presented any symptoms of joint disease. Of course, we cannot rule out early synovitis in symptomless individuals, although results from other studies show a rather late involvement of the synovia in the antibody-positive patients with joint complains [27].

The functional effects of Flt3L are mediated and occur through interaction with Fms-like tyrosine kinase 3, its receptor tyrosine kinase. Fms-like tyrosine kinase 3/Flt3L signaling has recently been shown critical for the development of early B-cell progenitors and dendritic cells, and for the expansion of induced regulatory T cells [10,28]. Blockade of Flt3L signaling using a small-molecule Fms-like tyrosine kinase 3 inhibitor ameliorates antigen-induced arthritis [29]. The experimental analysis of common biological processes linked high expression of survivin and Flt3L in RA patients and showed that Fms-like tyrosine kinase 3/Flt3L signaling was required for the expression of survivin *in vivo*[15]. In this case, the expression of survivin in leukocytes would appear as a downstream event of Flt3L signaling, and is in contradiction to the present observation of survivin as a predictor of disease development. Interestingly, survivin is increased in the preclinical phase of RA and showed association with another established predictor of RA, anti-CCP antibodies. Additionally, survivin was associated with the pattern of regulatory cytokines (IL-9, IL-12) already at the presymptomatic stage of RA and potentially supported formation of T-regulatory cells and proinflammatory Th1 and Th17 cell subsets with known pathogenic importance for RA [30,31].

Our recent study showed that the pattern of cytokines could be helpful in distinguishing presymptomatic individuals from controls or RA patients [5]. In the presymptomatic individuals, the RA-specific autoantibody production, including anti-CCP and IgM-RF, was strongly related to the T-cell cytokines. These cytokines are essential for the control of antibody production in the steps of engagement of antigen-presenting macrophages and B cells (that is, IL-13 and IL-4 [32]), for the maturation of induced T-regulatory cells and Th17 cells (that is, IL-9 [30]), and for the differentiation of follicular B cells and formation of germinal centers (that is, IL-4 [33]).

There are two major limitations within this study: a relatively small size of the tested cohort, and a broad variation of the time interval between the blood samples collected before the onset of the disease and the RA diagnosis. These samples were not collected on a regular basis but were retrieved from the existing bank of blood donors. Stratification of the studied subjects reduced the numbers in each group and made additional complications for the detection of statistical significance.

## Conclusions

In summary, increased levels of survivin in patients were detected years before the onset of RA symptoms, particularly in those individuals positive for anti-CCP antibodies. The high levels of survivin appear to be more related to the cytokines produced by the Th1 and Th17 subsets. These results suggest a role for survivin at the early presymptomatic stages of RA.

## Abbreviations

anti-CCP: anti-cyclic citrullinated peptide antibodies; ELISA: enzyme-linked immunosorbent assay; Flt3L: Fms-like tyrosine kinase 3 ligand; IL: interleukin; RA: rheumatoid arthritis; Th: T-helper type.

## Competing interests

The authors declare that they have no competing interests.

## Authors’ contributions

MBo contributed to the design and interpretation of the data and was involved in drafting the manuscript. MBr analyzed and interpreted the data, and was involved in drafting the manuscript. ME analyzed and interpreted the data, and was involved in drafting the manuscript. SRD contributed to the design of the study, analyzed and interpreted the data, and was involved in drafting the manuscript. All authors gave their final approval to the version to be published.
